# What Do Patients and Their Carers Do to Support the Safety of Cancer Treatment and Care? A Scoping Review

**DOI:** 10.1097/PTS.0000000000001031

**Published:** 2022-06-14

**Authors:** Darci Tillbrook, Kate Absolom, Laura Sheard, Ruth Baxter, Jane K. O’Hara

**Affiliations:** From the ∗ University of Leeds, Leeds; † Bradford Institute of Health Research, Bradford, United Kingdom; ‡ University of York, York.

**Keywords:** patient safety, resilience, patient involvement, resilience healthcare, safety-ii, cancer care

## Abstract

Cancer patients and their carers face a multitude of challenges in the treatment journey; the full scope of how they are involved in promoting safety and supporting resilient healthcare is not known.

**Objective:**

The study aimed to undertake a scoping review to explore, document, and understand existing research, which explores what cancer patients and their carers do to support the safety of their treatment and care.

**Design:**

This scoping review followed the Preferred Reporting Items for Systematic Reviews and Meta-Analyses Extension for Scoping Reviews guidelines. Five online databases were searched from 2000 to 2021 to identify primary literature exploring perspectives on patient and caregiver involvement in maintaining their safety during cancer care. Narrative synthesis was then conducted on the included literature.

**Results:**

Of the 1582 results generated from the initial search, 16 studies were included in the review. Most consisted of qualitative semistructured interviews with patients, as well as carers and healthcare professionals (HCP). Four narrative themes were identified: patient perception of safety and their involvement; patients take charge of their own care and well-being; safety as a dynamic collective responsibility; and carers are an undersupported asset. Patients and their carers involve themselves in a variety of behaviors in physical care, well-being, communication, and care coordination to ensure safety and support system resilience. This review adds a novel perspective on cancer patient and caregiver involvement in supporting resilient healthcare.

**Conclusions:**

Patients and their carers play an important role in promoting safe cancer care and healthcare system resilience. Further research is recommended to realize the full extent of the system gaps encountered and mediated by patients and their carers.

Patients receiving cancer treatment can face a multitude of safety risks. As high as 34% of cancer patients experience one or more adverse events, a much higher rate in comparison with the general patient population.^1,2^ In addition to undergoing physiologically toxic and complex care, cancer patients often face navigating transitions between multiple treatment environments and managing communication between different healthcare professionals (HCP) across long periods, which can create misunderstandings and variable standards of care.^3,4^ Although there is limited literature exploring the extent of errors and adverse events, it is known that a large proportion experienced by cancer patients were potentially preventable or mitigable.^2,5^ In addition, much of current safety research focuses on specific adverse events, which does not account for the dynamic experiences of cancer patients or the adaptability or capacity of the healthcare system (known as “resilient healthcare” theory).^6,7^ For example, patients with similar diagnoses often have differing treatment experiences, and it is the response of the healthcare system, which provides adequate care.

Across healthcare services, it is emerging that patient safety is influenced by the “resilience” of the system. The ability to respond, monitor, anticipate, and learn impacts the standard of care patients receive, and the “gaps” that are built in, or arise during care delivery can negatively impact patients.^3,6^ Furthermore, evidence is emerging that the repercussions of these gaps often depend on the adaptability of the HCPs involved (to, e.g., accommodate dynamic care priorities).^3,8^

Gaps within or between care services are also often bridged by patients and their carers (a term used to refer to family members, friends, or unrelated persons who support a patient through assisting with care and/or advocacy).^9^ Although patient and caregiver involvement in patient care has long been recognized, their role in promoting healthcare system resilience is only beginning to be explored empirically. Patients and carers have been described as “scaffolding” the healthcare system, by stepping in and supporting the system to function effectively.^9^ Such behaviors include following up results and informing HCPs of medication changes.^9,10^ Patients and carers also engage in error monitoring behaviors, such as notifying staff of deviations in expected treatment procedures, which further support system resilience and patient safety.^11^

Patient safety, which is defined as “the absence of preventable harm to a patient during the process of healthcare,”^12^ is not a well-understood concept among patients.^13^ Despite this, patients and carers have positive attitudes toward their involvement in ensuring safety.^14^ However, delegating responsibilities to or making assumptions about patients and carers can create burdens of care.^15^ When considered cumulatively, cancer patients and carers must navigate and overcome multiple gaps and challenges throughout the care journey. However, the full scope of behaviors that patients and carers initiate to support patient safety and system resilience is not known. In addition, the patient perception of these roles and how they interact with each other to support the patient and the resilience of the healthcare system have not been considered previously in tandem. This review aims to understand what cancer patients and carers do to support the safety of cancer treatment and care. The following research questions were devised to guide the review:

- How do patients and carers perceive their role within the treatment and care process?
- What aspects of care are patients and carers involved in?
- Do patients have different roles from the role of carers?
- Are safety responsibilities divided between patients, carers, and HCPs, and if so, how?
- How does the involvement of patients and their carers support the safety of their care and the service?

## METHODS

### Protocol

A protocol was drafted using the Preferred Reporting Items for Systematic Reviews and Meta-Analyses Extension for Scoping Reviews^16^ by one researcher (D.T.) and was reviewed by the rest of the research team (J.O.H., K.A., L.S., R.B.). A scoping review was deemed most appropriate in fulfilling the exploratory nature of the review aims.^17^

### Inclusion Criteria

The following inclusion criteria were applied to the search results:

- Literature was published between 2000 and 2021
- Literature focused on patient and caregiver experiences
- Literature described only experiences of normal practice
- Literature focused on patients older than 18 years and in nonpalliative care
- Literature was published in English
- Literature contained primary data and was peer reviewed

### Information Sources

Five databases (CINAHL, MEDLINE, PsycINFO, Web of Science, Embase) were searched by D.T. between March and June 2020; a revised search was conducted in January 2021. The search strategy was drafted by D.T. with guidance from an experienced academic librarian. All searches were recorded. Reference lists of included articles were checked for relevant literature.

### Search

The search strategy was separated into 3 concepts: (i) patients, carers, and their experiences; (ii) the safety of treatment; and (iii) cancer care. A concept table is in Appendix 1, http://links.lww.com/JPS/A515 and an example search is in Appendix 2, http://links.lww.com/JPS/A515.

### Selection of Sources of Evidence

**FIGURE 1:**
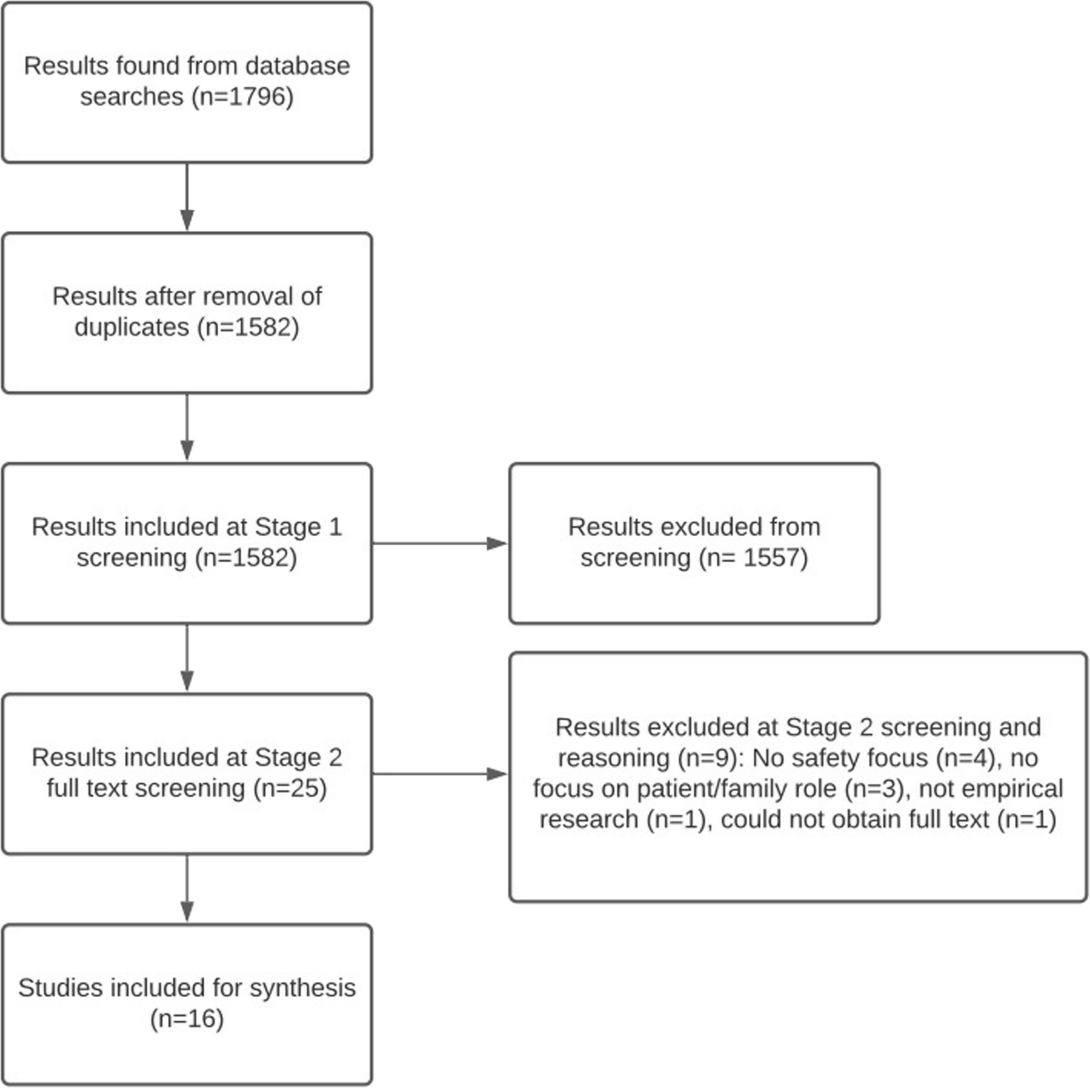
Flowchart depicting the process of selecting sources of evidence.

Two stages of screening were conducted. In stage 1, titles and abstracts were checked for relevance. Unclear literature was retained. In stage 2, the full text of literature included from stage 1 was reviewed. Stage 1 and 2 screening was conducted by D.T., with a randomized subsample (stage 1 [10%, n = 300], stage 2 [20%, n = 4]) of each stage results independently reviewed by J.O.H. and K.A. There were few differences between the reviewers in inclusion decisions, with only 1.7% (n = 5) of studies from stage 1 and no disagreements in stage 2. All disagreements were discussed by the 3 reviewers and resolved. Any uncertainties found by D.T. in either stage were also discussed and resolved (Fig. 1).

### Data Charting Process

A data charting form was developed by D.T. and reviewed by the research team. The form was based on the Joanna Brigg Institute Template Source of Evidence, Characteristics and Results Extraction Checklist,^18^ with modifications to collect information relevant to this review. Headings were formulated from characteristic data (e.g., data collection, summary of patient role). The full list is in Appendix 3, http://links.lww.com/JPS/A515.

### Synthesis of Results

Textual narrative synthesis was conducted, which collates the results in a homogenous manner, while maintaining contextual factors.^19^ Literature was examined for similarities and differences, the identification of which formed the basis of “themes,” which aggregated findings relevant to the review aims.

## RESULTS

### Selection of Sources of Evidence

#### Characteristics of Individual Sources of Evidence

**FIGURE 2:**
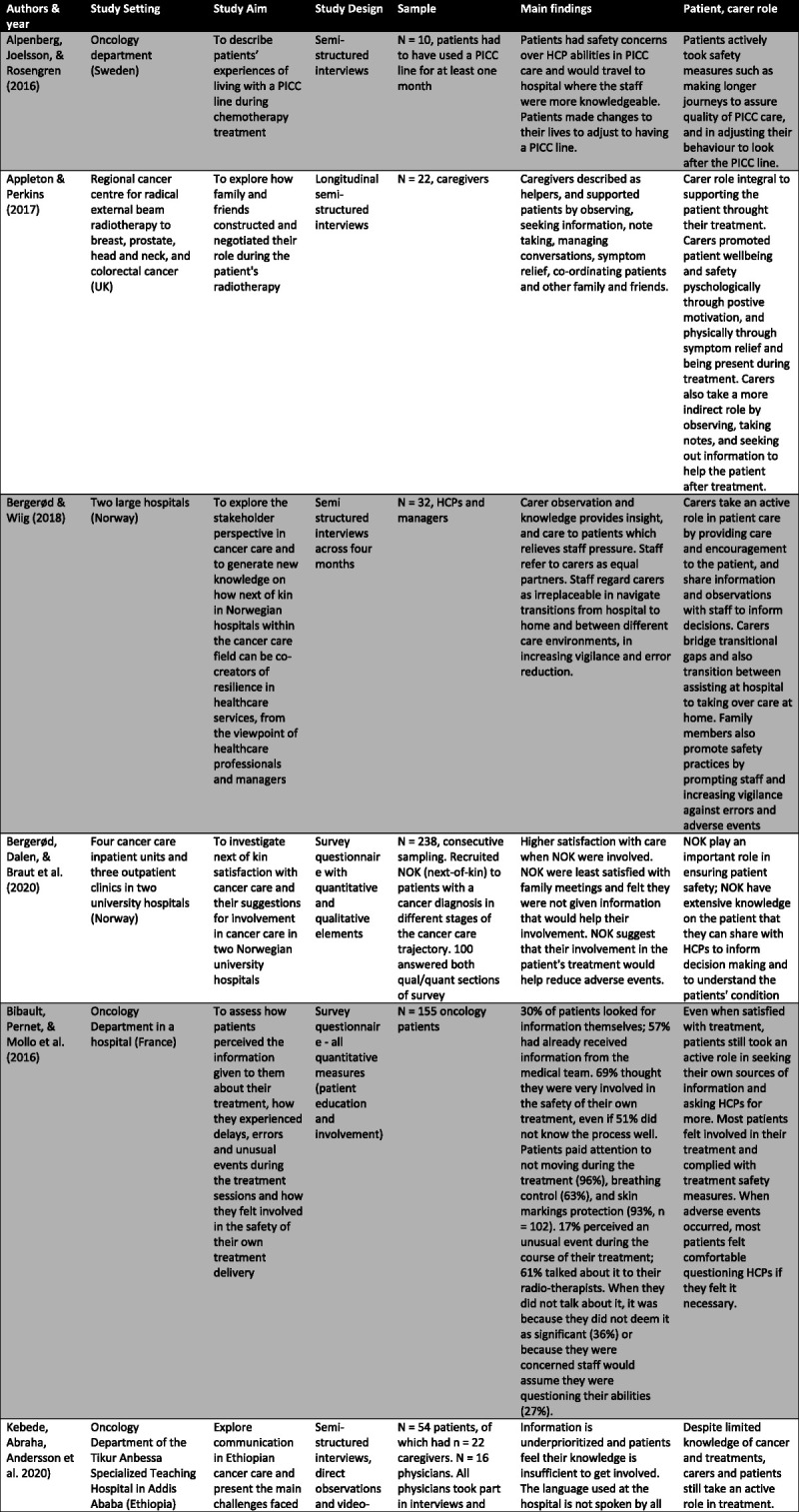
Table of study characteristics.

Specific characteristics were selected and charted separately as relevant to the aims of this study and presented in Figure 2.

**Figure:**
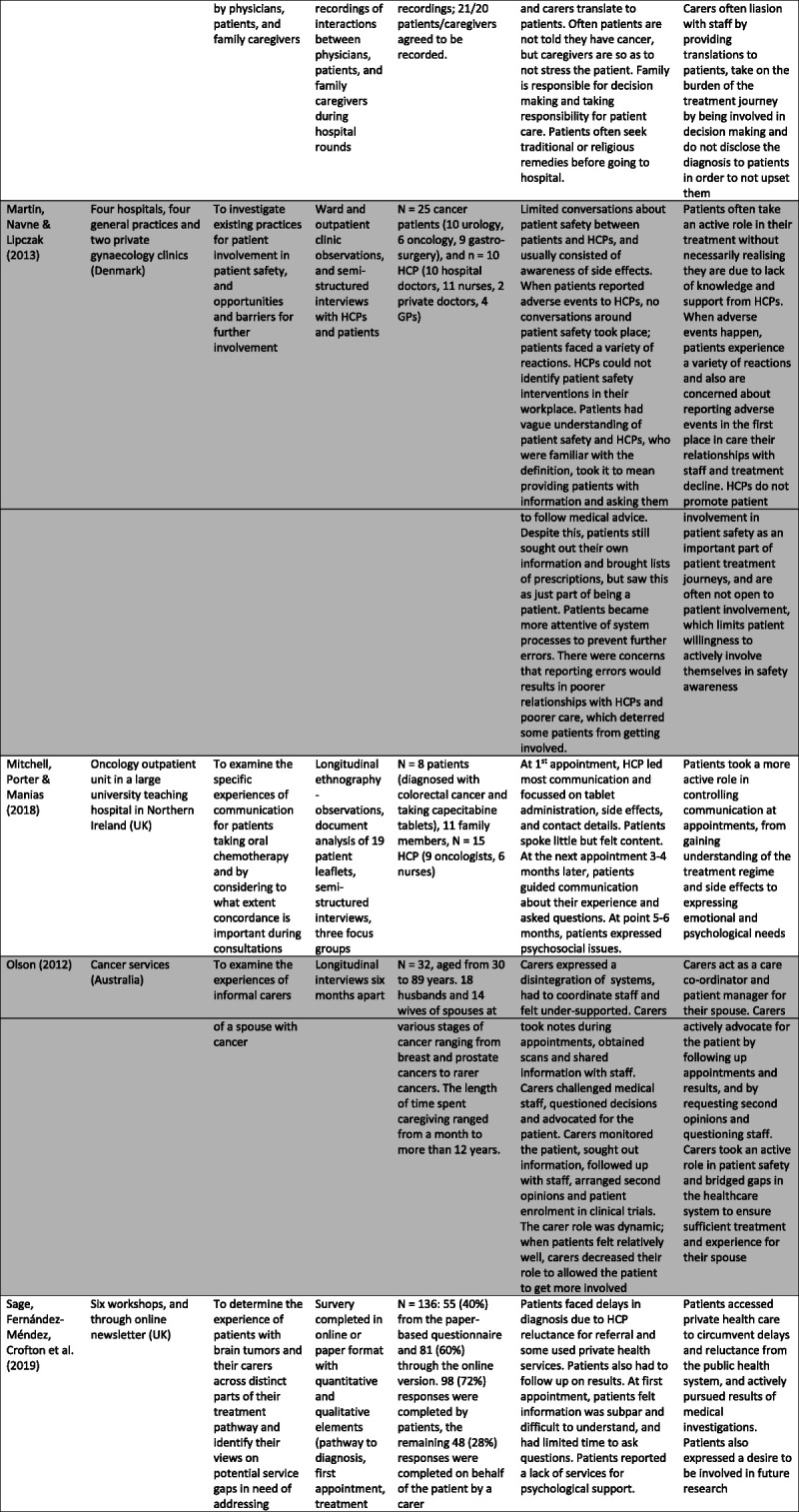

**Figure:**
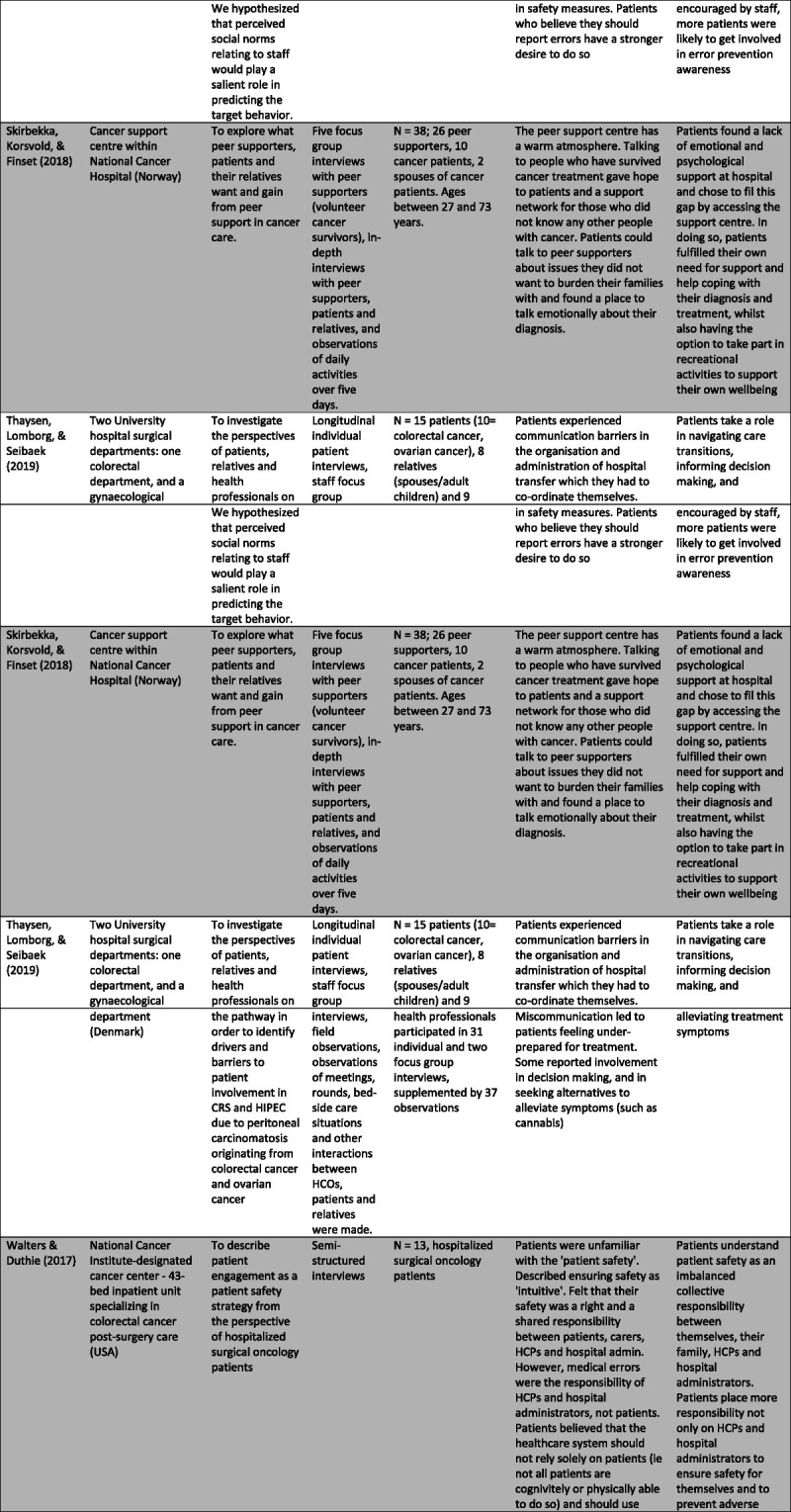

**Figure:**
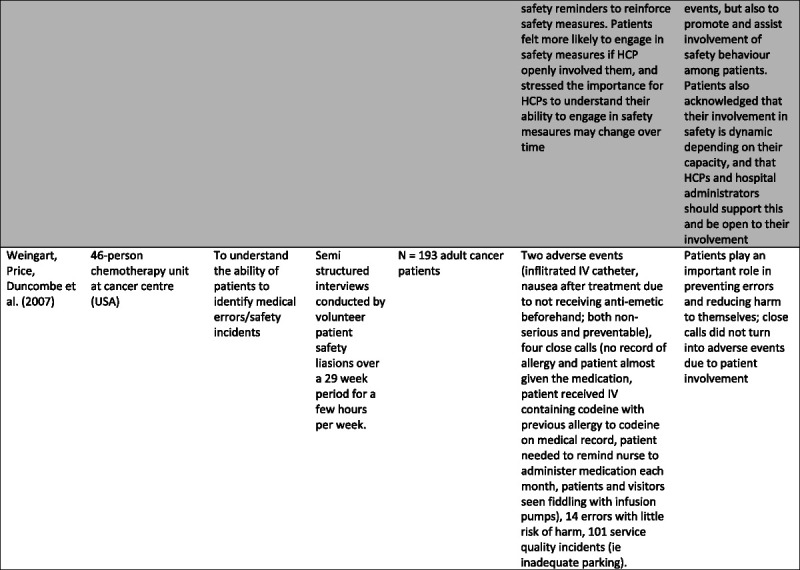

## FINDINGS

### Characteristics of Included Studies

Sixteen studies were selected for analysis.^4,11,13,20–32^ Most studies took place in European countries (n = 12). In addition, the majority (n = 13) took place in a treatment setting.

Twelve studies used qualitative methodologies,^4,13,20–22,25–27,29–32^ two adopted a mixed-methods approach,^23,28^ and the remaining two purely quantitative methodology.^11,24^ Most studies conducted semistructured interviews with individuals (n = 8),^4,13,20–22,25,27,32^ focus groups (n = 1),^29^ or both (n = 3).^26,30,31^ One study also incorporated unstructured interviews into its methodology.^29^ Of these studies, five conducted longitudinal interviews.^21,22,26,27,31^ In addition, ethnographic methods were used including direct observations (n = 5),^4,25,26,30,31^ video recordings (n = 1),^25^ and document analysis (n = 1).^26^

The four studies using a mixed-method or quantitative approach all administered questionnaires.^11,23,24,28^ Two examined patient and caregiver satisfaction with care and areas of improvement,^23,28^ while the remaining two explored patient motivators and predictors for involvement in safety behaviors.^11,24^ Due to the small number of quantitative papers, the findings were woven into the wider themes, which emerged from the qualitative studies to support and strengthen the analysis.

Of all sixteen studies, eleven sampled patients,^4,11,13,20,24–26,28,30–32^ eight sampled carers,^21,23,25–27,29–31^ and five sampled HCPs.^4,23,25,26,31^ All studies that sampled carers were identified as members of the patient’s family and one included an unrelated friend.^21^ Of studies involving staff, three included oncologists,^4,22,26^ three nurses,^4,22,26^ one recruited general practitioners,^4^ one sampled surgeons,^25^ and one did not specify.^31^

### Narrative Synthesis

Four themes were identified: (i) patient perception of safety and their involvement; (ii) the patient role in care and well-being; (iii) safety as a dynamic collective responsibility; and (iv) carers are an unsupported asset.

#### Patient Perception of Safety and Their Involvement

Two studies described good patient understanding of “safety,” but vague awareness of “patient safety.”^4,13^ Furthermore, patient interviews and observations found that HCPs did not talk to patients specifically about it and predominately focused on treatment adverse effects and compliance.^4^ When asked, HCPs concluded that patient safety was promoting compliance.^4^ There is limited acknowledgement of the importance of communicating “patient safety” to patients and a lack of system-level promotion.

Despite a lack of understanding of “patient safety” as a concept, many patients still reported involvement even if they did not know the treatment or process well.^24^ Patients generally did not seem to view their involvement as a “role”; many described their involvement in safety as just “part of being a patient,”^4^ or that their behavior was intuitive, or common sense.^13^

#### Patients’ Role in Care and Well-being

Patients reported involvement in communicative, physical, and psychological aspects of care as well as error prevention.

##### Communication

The degree to which patients received information was equivocal, with 2 articles reporting information to be adequate^24,26^ and 3 articles reporting suboptimal access.^25,28,31^ In addition, patients sought out sources of information^4^ and approached HCPs.^24^ Patients also asked questions^26^ and guided decision making.^31^ Patients directly influenced the safety of the treatment pathway by following up about results^28^ and coordinating hospital transfers.^31^

##### Physical Care

Two studies reported patient involvement in physical care.^20,24^ In one study, most patients stayed still during radiotherapy treatment (96%), tended to their skin markings (93%), and controlled their breathing (63%), all behaviors promoting safety during and after treatment.^24^ In addition, patients fitted with peripherally inserted central catheter lines supported care at home by covering or raising their arm in the shower to avoid water contact.^20^ Also, some patients reported inconsistencies in HCPs visiting their homes to provide PICC line care and to avoid harm and took long journeys to hospital to access care from experienced staff.^20^

##### Psychological Well-being

Four studies reported patient involvement in well-being.^26,28,30,31^ One study found that at 5 to 6 months after commencing treatment, patients spoke with HCPs about psychosocial issues including mood management.^26^ Two studies^28,30^ found that patients lacked psychological support. Some found ways of finding support, with peer support centers regarded as a welcoming place.^30^ Some patients accessed alternative methods such as cannabis use to relieve symptoms.^31^

##### Obstruction of Care and Adverse Events

One study found that patients obstructed care.^25^ In Ethiopia, the general population is not knowledgeable about cancer and those who develop it often seek advice from religious leaders instead of clinicians.^25^ Such actions can lead to diagnosis delays and worse prognoses.

Four studies reported adverse patient events.^4,11,24,32^ One study found that 17% of patients experienced an “unusual event”; 61% of these patients spoke to an HCP.^24^ Those who did not deemed the event trivial or believed staff competency would be questioned.^24^ This is a legitimate concern; patients who report adverse events to HCPs can face hostility.^4^

In addition, patients mitigate adverse events. In one study, 2 “close call” events were de-escalated by patients.^32^ One patient was treated with medication they were allergic to and alerted a nurse, and another reminded a nurse to administer a monthly medication. In both cases, patients alleviated safety risks. Those who experienced adverse events also reported higher vigilance to prevent future errors.^4^

##### Barriers

Three studies described obstacles to involvement.^4,11,25^ Some lacked information to involve themselves,^25^ while others felt that management was “out of their control” and there was no expectation for their involvement.^11^ Patients also worried that if they did report errors, staff relationships would sour and result in jeopardization of their standard of care.^4^

#### Safety as a Dynamic Collective Responsibility

Three studies presented patients’ views regarding responsibility for patient safety.^4,11,13^ One study reported that patients believe patient involvement in patient safety is a right but also a shared responsibility between themselves, carers, and HCPs.^13^ The distribution of responsibility between each party is, however, unequal. Patients believe that HCPs have a “duty of care” to shoulder serious responsibilities, such as medical errors.^13^ In addition, staff have a role in promoting patient involvement in safety. Three studies found that patients felt more likely to engage in safety behaviors when staff encouraged it.^4,11,13^ Indeed, those that felt staff did not expect patient involvement were less inclined.^4,11^ Patients further emphasized the importance of split responsibility as patient ability may not be ever-present; not all patients have the physical or cognitive capacity to be involved in safety behaviors.^13^

#### Carers Are an Undersupported Asset

Six studies reported on the role of carers.^21–23,25,27,29^ One study reported that carers did not see themselves at “carers”; patients were cared for by the healthcare system, and carers were helpers.^21^ However, carers reported being involved in physical care to relieve pressure from staff.^22^ Another study reported carers keeping notes and motivating patients.^21^ In addition, patients in Ethiopia are linguistically diverse and rely on carers to facilitate communication with HCPs.^25^

Carers maneuver patients through the healthcare system.^27^ Carers described themselves as patient advocates and “safety nets.”^21,27,29^ Some stressed their role as essential to patient survival.^29^ Carers spoke of questioning staff and arranging enrollment in clinical trials.^27^ In Ethiopia, diagnoses were often not disclosed to patients to avoid upset; their families took over decision making.^25^

In one study, staff described carers as equals to HCP in making patient-tailored decisions.^22^ Carers felt that their involvement could reduce adverse events.^23^ Carers were described as irreplaceable in supporting patients through care transitions and in prompting staff to be vigilant of errors.^22^ However, carers often felt unprepared and underinformed.^23,29^ Furthermore, some carers felt unable to disengage from their role and were on constant guard.^29^ However, when involvement was supported by staff, carers reported higher satisfaction with patient care.^23^ Carers also acknowledged their role as dynamic; when patients felt relatively well, carers reduced responsibilities to allow patient involvement.^27^

## DISCUSSION

This review explored what patients and their carers do to support the safety of cancer care and healthcare system resilience. We found that patients and carers engage in a variety of safety-promoting, error-preventing behaviors. Barriers to involvement were also identified, as well as obstructions to care and perspectives on safety responsibilities. To the author’s knowledge, this is the first review to specifically explore the activities undertaken by cancer patients and carers that contributes to the safety of care. Our findings raise several important issues that will now be considered in turn.

### Is Supporting Safety a “Patient Role”?

Few studies explored what “patient safety” actually meant to patients, although it was clear that it was an uncertain concept to most.^4,13^ Patients also often dismissed their involvement as a “role,” considering their behaviors more instinctual and part of their capacity as a patient, despite not always knowing treatment processes well.^4,13,24^ Included studies provided substantial accounts of how patients filled in system gaps^4,20,24–26,28,30,31^ and monitored the system to prevent errors.^4,11,24^ Patients reported having a strong desire to be involved in their own safety with support and good relations with carers and HCPs and to be part of a “coalition of care.”^13^ Patients were directly involved in physical care, communication, and error monitoring and also organized their own psychological care. After experiencing errors, patients became more vigilant of the system to prevent further safety issues.^4^ Such evidence is consistent with the emerging concept of patients and carers as “scaffolding” services; this conception is proposed by the authors to describe activity, which is undertaken (often unseen) by patients and carers, that not only supports their own safety but also, in effect, acts as a further support—or “scaffold”—for system-level safety outcomes.^9^

This scaffolding role does not always seem to be equally distributed across all aspects of care, however. Despite some patients expressing a need for psychological support,^26,28,30^ only one study reported on well-being services accessed by patients.^30^ Studies with staff participants also did not find any mental health resources being accessed by or recommended to patients. This is particularly concerning because various reviews have found access to mental health services and interventions to be critical to cancer patients^33,34^; indeed, research has estimated more than half of cancer patients experience depression.^35,36^ In addition, while patients expected to undertake a share of responsibility for their care, many felt excluded and outside the treatment management sphere.^11^ Finally, patients in Ethiopia unintentionally obstructed care because of misinformation.^25^ Indeed, no studies explored staff perspectives on patient involvement; staff did, however, commend caregiver involvement.^22,23^

### Do Carers “Scaffold” the “Scaffolders”? The Caregiver Role

In contrast to patients, carers recognize and acknowledge their role as a safety net for patients and essential to ensuring quality of care.^21,27,29^ Carers acted not only as a mediator between patients and staff but also as an advocate for patients. Furthermore, carers in some cultures take on full responsibility for care decisions.^25^ Carers questioned staff decisions but also cared for patients both in hospital and postdischarge.^22,27^ Carers seemed to have conflicting views on their role and the system—some reasoned that the patient is cared for by the healthcare system and they are “just helpers,”^21^ while others experienced a broken system that they had to navigate for the patient and provide physical care.^27^ Regardless, carers could not express the importance of their involvement more strongly, and staff considered them equal partners in patient care.^22,29^ In this sense, carers scaffold the healthcare system in conjunction with patients and with encouragement from staff.^9^ Carers directly contribute to the resilience of the healthcare system by supporting error prevention and supplementing patient care to avoid gaps in treatment.^6^ The essential role carers provide is one they take on despite it postponing their own lives, and one they cannot disengage from.^21,29^ However, although no studies explored patients’ views on caregiver involvement, carers did understand their responsibilities varied depending on patient capacity and staff engagement.^13^

### Who Is Responsible for Patient Safety?

Patient involvement in patient safety is a right, and patients wish to be part of a “coalition of care.”^13^ Patients and carers felt responsible for using “common sense” and that HCPs are responsible for circumventing medical errors and top-down processes.^4,11,13^ In addition, patients must receive culturally appropriate information about these diagnoses to avoid unintentionally obstructing care.^25^ However, patient capacity was also recognized as influencing patient involvement in safety and support by HCPs to engage in safety and resilience supporting behaviors.^4,11,13^ Patients and carers should not be “burdened” by their involvement or HCP expectations, because this delegation of responsibility could lead to poorer safety outcomes.^15^

#### Limitations

The definition of patient safety does not define criteria for what activities qualify as such.^12^ Therefore, this review may have omitted unexplored behaviors that patients engage in. In addition, only one study was included in this review that relates to healthcare in a low-income country, meaning that conclusions in such healthcare contexts could not therefore be made. Finally, gray literature was not included in this review, which may have been relevant to the review aims.

#### Implications

Patients and carers occupy a unique position both “inside” and “outside” healthcare system pathways.^9^ The reviewed literature suggests that patients and carers identify gaps in cancer care and take initiative to minimize disruption and prevent harm. These actions are not limited to any category of behaviors or specific treatments and highlight the adaptability of patients and carers to “step in” not only when the resilience of systems is suboptimal but also in maintaining day-to-day resilience.

The findings of this review support the “scaffolding” role concept of patients and carers’ interaction with the healthcare system.^9^ In addition, this review links to emerging literature that patient and caregiver involvement is crucial to support resilience in healthcare systems (see the study by Guise et al^37^). Cancer patients are a unique patient population with distinct experiences of multiple care transitions (chemotherapy, radiotherapy, outpatient services, home-based care, to name a few)^4^ and are a novel representation of how patients safeguard themselves and navigate fluctuating resilience in healthcare systems. Furthermore, this review specifically contributes to the limited research pool on cancer patient and caregiver involvement in healthcare system resilience (see the study by Bergerød et al^22^). Such research is important to understand the unique gaps that exist in cancer care, how they are experienced by patients and carers, and how they attempt to mitigate negative consequences to support system resilience. Future research should explore patient and caregiver perspective on the scope of existing gaps in the cancer care pathway and their roles in promoting safety.

#### Conclusions

Patients and carers perform an important role in promoting healthcare system resilience and supporting safe cancer care. The reviewed literature describes the scope of care and error prevention activities that are undertaken by patient and carers. This review provides a foundational understanding for future novel research into cancer patient and caregiver involvement in supporting system resilience and recommends further exploration into system gaps and the role of patients and carers in adapting to inconsistent care.
